# Comparing Simultaneous and Pointwise Confidence Intervals for Hydrological Processes

**DOI:** 10.1371/journal.pone.0147505

**Published:** 2016-02-01

**Authors:** Mario Francisco-Fernández, Alejandro Quintela-del-Río

**Affiliations:** Department of Mathematics, Faculty of Computer Science, Universidade da Coruña, Campus de Elviña, s/n, A Coruña 15071, Spain; University of East Piedmont, ITALY

## Abstract

Distribution function estimation of the random variable of river flow is an important problem in hydrology. This issue is directly related to quantile estimation, and consequently to return level prediction. The estimation process can be complemented with the construction of confidence intervals (CIs) to perform a probabilistic assessment of the different variables and/or estimated functions. In this work, several methods for constructing CIs using bootstrap techniques, and parametric and nonparametric procedures in the estimation process are studied and compared. In the case that the target is the joint estimation of a vector of values, some new corrections to obtain joint coverage probabilities closer to the corresponding nominal values are also presented. A comprehensive simulation study compares the different approaches, and the application of the different procedures to real data sets from four rivers in the United States and one in Spain complete the paper.

## Introduction

Studies about river flows or, in general, hydrological processes are based on available records. Many times, the amount, accuracy and representativeness of the records are not enough to achieve an adequate reliability in results. Usually, there is a considerable uncertainty in measuring extreme values. In order to increase the accuracy of the estimates, several procedures of flood frequency analysis employing all the geographical and hydrological information available in the region of interest have been proposed. Some of the most handled methods are based in the estimation of quantiles. A *p*-quantile *x*_*p*_ of a random variable *X* is defined as the value which is exceeded with probability *p*, that is *P*(*X* > *x*_*p*_) = *p* [1]. In a hydrological context, this quantity may correspond to design values of environmental loads (waves, snow), river discharges and flood levels. Quantile and return level notions are closely related. If the variable *X* measures the river flow, the return level for a given period *T* is the quantile *x*_*T*_ with probability 1/*T* [2]. Formal definitions of the return level and return period concepts are given in the following section.

Quantile estimation is a valuable issue in decision making in water-related models. It appears naturally as a secondary factor in the estimation problem of *F*, the cumulative distribution function (CDF) of the variable *X*. Usually, given a data set, the CDF is fitted assuming that this function belongs to a known parametric family. Then, the corresponding unknown parameters have to be estimated [3]. This parametric scheme has been widely investigated in the specialized literature and, in recent years, efforts have been made in developing more accurate and robust estimation techniques [4]. Note that to apply these parametric approaches, a statistical model for the variable under study has to be assumed, which can represent an important drawback of this methodology if model assumptions are violated. On the other hand, nonparametric techniques have been employed to deal with hydrological problems in the last decades. Nonparametric estimation uses the information provided by the recorded data to construct directly estimators of the functions of interest, supposing only general assumptions about the random variable in study. These kind of proposals are normally well adapted to the more irregular shapes found in practice for hydrological variables. Applications of the CDF estimation in this setting are presented, for instance, in [1, 5, 6] and [7].

In both cases (parametric and nonparametric estimation), the evaluation of the estimator variations can be made by computing an assessment of their uncertainty. Some significant research on uncertainty analysis in a hydrological context has been performed in, for example, [8, 9], and references therein. In the present research, we are mainly interested in the study of the statistical uncertainty related with the prediction of statistical measures of interest (quantiles) of certain random variables [10]. This can be carried out calculating confidence intervals (CIs) for these quantiles or return levels. The proper estimation of these CIs provides hydrology experts with a robust tool in the risk evaluation and management strategies. Return level overestimation clearly supposes an increase of the costs related with monitoring and life protection. On the contrary, underestimation of return leves could lead to increase damages and associated costs, with the consequent losses for insurance companies. CI estimation for quantiles was previously considered in, among others, [6, 10–13]. Other types of uncertainty include the randomness of the natural process or the uncertainty depending on the choice of a particular model [10] and are excluded from this study.

In hydrology, CIs are usually computed for given quantiles corresponding to fixed return levels. Given a high confidence level (95%, for instance), each one of these CIs will contain the corresponding unknown quantile with a high probability (0.95, for example). In other words, a single 0.95-CI for each one of the quantities of interest is computed and, in this sense, they are called pointwise CIs. However, if the target is the estimation of a vector of values (such as the CDF or the return level function evaluated in a grid of points, for instance), more accurate than computing the corresponding pointwise CIs for each one of the quantities of interest is to construct simultaneous CIs (also called a confidence band). Simultaneous CIs are a group of CIs (a CI for each one of the quantities of interest) designed to jointly contain this vector of unknown values with a prescribed high probability (95%, for instance). In this way, it would be possible to make a joint probabilistic assessment for some quantiles of the corresponding CDF. Because simultaneous CIs include information on model reliability, they are are much more informative than pointwise CIs [14].

Different techniques have been proposed to produce CIs. In a parametric framework and assuming a certain distribution for the variable under study, exact or approximate CIs could be constructed, using maximum likelihood theory or the maximum product of spacings method (proposed in [15]). Nevertheless, in some practical situations, these approximations can be quite inaccurate (some numerical examples of this issue can be found in, for example, [16–18]), or it could be necessary to employ theoretical calculations and manipulation of complicated forms of estimators. Moreover, in a nonparametric setting, these approaches cannot be applied and it is necessary to use other proposals. The present research focuses on the use of bootstrap methods to construct CIs. These techniques allow to overcome some of these problems. In general, bootstrap [16] is a resampling technique that attempts to estimate the sampling distribution of a population, and to determine the accuracy of statistics, by drawing new samples from the original data. Depending on the resampling process used, the method is called nonparametric bootstrap, when the new samples are obtained with replacement from the original data, or parametric bootstrap, when they are randomly generated from a parametric model (distribution) fitted to the data. Under certain probabilistic condition (readers are referred to the first chapter of [19], where the theoretical conditions required for this method to work are briefly described), bootstrap methods can produce more accurate and reliable results than the traditional methods based on asymptotic approximations (using central limit theorems, for instance). This is specially true in situations when the distribution of the variables under study are unknown and the distribution of these variables is far from normality [20]. Nowadays, as a computer-aided statistical technique, bootstrap is largely used in many fields of applied statistics such as medicine, biostatistics, or hydrology [21–23].

Different approaches producing good approximate bootstrap CIs have been proposed. These include, among others, standard normal bootstrap CIs, percentile bootstrap CIs, basic bootstrap CIs, bootstrap-*t* CIs or corrections of the previous methods using, for example, the Bias Corrected and accelerated (BCa) technique [17, 24]. Some of these procedures to construct CIs have been used to compare parametric and nonparametric bootstrap approaches for extreme value distributions in [12, 13]. The main conclusions of these papers is that the differences among the different types of bootstrap CIs (percentile, bootstrap-*t* and BCa) are usually smaller than those between the parametric and nonparametric versions of bootstrap. Furthermore, in [12, 13], it is also concluded that parametric bootstrap should be preferred for small to moderate sample sizes, and when a suitable distribution for the data is known or can be assumed.

When the estimation target is a function, to construct a 1 − *α* simultaneous CI, that is, a confidence band containing the whole curve with a prescribed probability of 1 − *α*, one can think of computing the 1 − *α* pointwise CIs in a grid of selected points using one of the previous techniques, and considering this set of CIs as the confidence band. It is clear that the probability that the whole curve is inside this band is smaller than the desired 1 − *α*. Some approximations have been proposed to address this problem, for example, the well-known Bonferroni correction [25]. In this research, simultaneous CIs constructed applying the Bonferroni method, and also using a correction of this approach trying to solve the well-known conservative feature of the Bonferroni technique are compared with synthetic data.

In the current paper, a comprehensive simulation study comparing different pointwise and simultaneous bootstrap CIs of the return level function and the CDF, mainly focusing on estimations obtained in large quantiles, are presented. As stated previously, the estimation of these values is a very interesting issue in hydrology. In the present research, nonparametric bootstrap methods are used in the resampling process, and parametric and nonparametric approaches are employed to estimate the functions of interest. This allows, on the one hand, comparing both estimation methodologies in this framework and, on the other hand, observing the influence of using pointwise or simultaneous CIs when the estimation target are the return levels and the CDF evaluated in large quantiles. The study is completed with the application of the bootstrap approaches to real flow data from US rivers previously used in [10], and to a data set from a Spanish river (Ebro river).

## Statistical methods

### Statistical functions

In hydrological applications, CDF estimation is considered an important problem due to the existence of relevant functions of interest directly related to it. One of these functions is the *probability of exceedance*. It gives, for a value *x*, the probability of occurrence of a flow larger than *x*. This information is quite significant in this framework. If *X* is the variable measuring the flow, the function returning the probabilities of exceedance is simply defined as

$$\begin{array}{c} R(x)=P(X>x)=1-F(x), \end{array}$$ (1)

with *F* the CDF of *X*. The *return period* or *recurrence interval* is directly associated with it. It is defined as the mean time interval between exceedances of a specific value *x*. This is important particularly when one is dealing with extreme events such as floods, droughts, and wind speeds. A formal definition of this concept, under some assumptions, is given, for example, in [26]. If *X*_*i*_ represents the flood in year *i*, with *i* = 1, 2, 3, …, and assuming that these variables are independent and identically distributed, the probability that the time interval $\tilde{T}$ between exceedances of a flood of magnitude *x* equals *n*, *n* = 1, 2, …, corresponds to the probability function of a geometric distribution, given by:

$$\begin{array}{ccc} P(\tilde{T}=n) & = & P\left(X_{1}<x\right)P\left(X_{2}<x\right)\cdots P\left(X_{n-1}<x\right)P\left(X_{n}>x\right)=\\ & = & {\left[P\left(X<x\right)\right]}^{n-1}P\left(X>x\right). \end{array}$$

Therefore, the return period, defined as the mean of $\tilde{T}$, is given by:

$$\begin{array}{c} RT\left(x\right)=\frac{1}{P(X>x)}=\frac{1}{1-F(x)}. \end{array}$$ (2)

In many practical problems, it is useful to estimate quantiles corresponding to certain values of the probability of exceedance. The *T* –*return level* is defined as the value that can be expected to be once exceeded during a *T* –period of time, that is

$$\begin{array}{c} RL\left(T\right)=x_{T}=F^{-1}\left(1-\frac{1}{T}\right). \end{array}$$ (3)

### Parametric estimation

A first approximation to estimate *F* consists in using a parametric methodology. If the empirical distribution function of a sample of data suggests that *F* may belong to a specific parametric family (Gaussian, Gamma, etc.), depending on a vector of parameters *θ*, one just has to estimate the unknown values of that vector of parameters to obtain a parametric estimator of the CDF. Denoting by $\hat{\theta }$ an estimator of *θ* obtained from a sample data (*X*_1_, *X*_2_, …, *X*_*n*_), an estimator of *F*, denoted by $F_{\hat{\theta }}$, is immediately derived. Obviously, from $F_{\hat{\theta }}$, parametric estimators of the functions given in Eqs (1), (2) and (3) are also easily constructed:

$$\begin{array}{c} R_{\hat{\theta }}\left(x\right)=1-F_{\hat{\theta }}\left(x\right),\;RT_{\hat{\theta }}\left(x\right)=\frac{1}{1-F_{\hat{\theta }}\left(x\right)}\;\text{and}\;RL_{\hat{\theta }}\left(T\right)=F_{\hat{\theta }}^{-1}\left(1-\frac{1}{T}\right). \end{array}$$ (4)

In this parametric context, if the variable *X* with distribution function *F* represents the maximum value of a quantity of interest measured in a specific period of time, classical extreme value theory uses the idea that, under certain regularity conditions [27], the limit of the distribution of *F* is the GEV distribution function. This function is considered to correspond to one of the following three families,

$$\begin{array}{c} F_{\theta }\left(x\right)=\left\{ \begin{array}{c} \exp\{-{\left[1+\gamma (x-\mu )/\sigma \right]}^{-1/\gamma }\},\\ 1+\gamma (x-\mu )/\sigma >0,\;\gamma \neq 0,\\ \exp\{-\exp[-(x-\mu )/\sigma ]\},\;\gamma =0. \end{array}\right. \end{array}$$ (5)

with *θ* = (*μ*, *σ*, *γ*). Here, *μ* is the location parameter, *σ* > 0 is the scale parameter, and *γ* is the shape parameter. The case of *γ* = 0 is named the Gumbel distribution.

### Nonparametric estimation

Parametric modeling traditionally used in hydrological processes, however, does not always provide good results. An initial hypothesis on the parametric model for the variable of an unknown function has to be assumed when using this type of approaches. Goodness-of-fit tests can be applied to check the validity of these models. However, violated assumptions of the parametric model or an incorrect estimation of its parameters may lead to a poor performance of the corresponding estimators. Additionally, anomalous events can lead to wrong results in these goodness-of-fit tests. Therefore, a nonparametric approximation can be useful in such situations. This methodology avoids the specification of a particular model to work with (such as the normal distribution, or a linear relation), and it is a valid alternative to the classical parametric techniques [28, 29]. Regarding the CDF, *F*, given a sample data (*X*_1_, …, *X*_*n*_), the well-known kernel CDF estimator [1] is defined at any point *x* of the domain of *F* as:

$$\begin{array}{c} {\hat{F}}_{h}\left(x\right)=n^{-1}\sum_{j=1}^{n}H\left(\frac{x-X_{j}}{h}\right), \end{array}$$ (6)

where *h* is the bandwidth or smoothing parameter, and $H\left(x\right)=\int_{-\infty }^{x}K\left(t\right)dt,$ with *K* a kernel function, usually a density function or a smooth function with some regularity conditions [29]. Based on definition (6), estimators of the functions given in Eqs (1), (2) and (3) can be also derived, substituting $F_{\hat{\theta }}\left(x\right)$ by ${\hat{F}}_{h}\left(x\right)$ in Eq (4).

Although the choice of the kernel function is of secondary importance, the smoothing parameter *h* plays a crucial role in the kernel estimator Eq (6). Small values for *h* make a highly variable estimator while big values produce a very smooth one [6]. Therefore, it is very important to use automatic bandwidth selection methods producing reliable estimators.

In the nonparametric distribution estimation framework, only plug-in and cross-validation approaches have been considered to select the bandwidth. In general, they have shown a good performance from a theoretical and practical point of view. Two different plug-in methods, developed by [30] and [31], can be cited. Cross-validation techniques have been investigated by [32], obtaining good experiences in practice. It can also be seen in [33] and references therein that, in distribution function estimation, similar results can be obtained in practice with both cross-validation and plug-in bandwidths. However, cross-validation has a clear drawback in terms of computing time. The cross-validation algorithm is an *O*(*n*^2^) in terms of asymptotic time complexity, while the plug-in method is only *O*(*n*); *n* being the sample size (see also [6], for more insight in bandwidth selection in distribution function estimation in hydrology). This concern is increased in the comparisons based on simulations, where the whole number of calculations are repeated a large number of times. Therefore, given that both bandwidth selection procedures (cross-validation and plug-in methods) seem to provide similar results in this framework, it is preferred to use the plug-in method of [31] in these experiments. Note that this plug-in approach has employed previously in related works, with good results [34]. In the recent paper of [33], the interested reader can find more theoretical details and an extended discussion on these two bandwidth selection methods for the nonparametric distribution function. Additionally, a free package, named Kerdiest, using the R software regarding these procedures was also developed.

The previous bandwidth selection methods provide global smoothing parameters. However, in situations with observations coming from a long-tailed density, a possibility to improve the behavior of nonparametric kernel estimators is to consider local bandwidths instead of a global smoothing parameter. There are several approaches to deal with this problem. In the nonparametric kernel density estimation framework, a solution is to use the so-called adaptive kernel estimator. The idea of this procedure is to combine features of the kernel and the nearest neighbor methods. It consists of constructing a kernel estimate with “bumps” or kernels located at the observed data points, but with different bandwidths from one point to another [35]. Some other authors have addressed the problem of estimating return levels or return periods for extremely large events using nonparametric estimators of these quantities with local bandwidths. For example, in a hydrological context, [36] or [1] discussed the use of variable or local bandwidths to address the extrapolation problem. A well-known drawback of the estimators using this kind of bandwidths is the possible increase of its variance, leading to similar mean squared errors to those obtained with global proposals. Additionally, local or adaptive bandwidth selection approaches add an extra computing time to the whole process and, for this reason, it was preferred not to use data-adaptive smoothing parameters in the present research. However, a deeper study of this issue could be carried out in a future research.

### Confidence intervals

In the previous section, different parametric and nonparametric estimators of some functions of interest in this framework were described. As pointed out, the CDF of the variable under study can be considered the key function for the rest of them, and it is reasonable to think that the properties of the CDF estimates can be extended to other functional estimates (exceedance, return levels, etc.). For this reason, the CDF is the main interest of this analysis. However, results concerning the return level function have also been obtained. The procedure to construct the CIs is basically the same for the CDF and the return level function. Hence, just the process for the CDF, *F*, is described. A similar analysis was carried out for the return levels given in Eq (3).

Given a point *x*_0_ of the *F* domain, $\hat{F}\left(x_{0}\right)$ denotes a general parametric or nonparametric estimator of *F*(*x*_0_). In many situations, a point estimate does not provide enough information about a value of interest (*F*(*x*_0_), in this case). A single number may not be as meaningful as an interval within which it would expected to find the value of this variable. As it is well-known, a (1 − *α*)-CI is a random interval, (*L*, *U*), containing the parameter of interest with a (high) probability 1 − *α*. For a specific sample realization (*x*_1_, …, *x*_*n*_), a numeric interval (*l*, *u*) is obtained, called a 100(1 − *α*)% CI, in the sense that drawing infinity samples and constructing the corresponding numeric intervals, the 100(1 − *α*)% of such intervals will contain the unknown parameter.

Among the different existing methods to compute CIs, several bootstrap approaches were employed in the present research. This resampling technique allows to obtain good approximate CIs in a relatively simple way. Next, the proposals used in this research are briefly described. In a first stage, pointwise bootstrap CIs for *F*(*x*_0_) are presented. Then, the issue of constructing simultaneous CIs is addressed, considering two algorithms trying to correct the possible bad performance when the previous pointwise methods are applied to obtain confidence bands.

#### Pointwise confidence intervals

In this Section, the approaches compared in this analysis, providing the (1 − *α*) pointwise bootstrap CIs for *F*(*x*_0_) are briefly described. In a practical situation, small values of *α* (*α* = 0.01 or 0.05, for instance) are usually employed. Readers are referred to [24] and [20] for theoretical properties and discussion of empirical performance of these methods.

**The standard normal bootstrap CI**. This is the simplest approach, but not necessarily the best. Suppose that $\hat{F}\left(x_{0}\right)$ is a parametric or nonparametric estimator of *F*(*x*_0_), and assume the standard error of the estimator is $\sigma (\hat{F}\left(x_{0}\right))$. Using the Central Limit Theorem and assuming that $\hat{F}\left(x_{0}\right)$ is an unbiased estimator of *F*(*x*_0_), an approximate (1 − *α*)-CI for *F*(*x*_0_) is
  $$\begin{array}{c} {}[\hat{F}\left(x_{0}\right)-z_{\alpha /2}\sigma \left(\hat{F}\left(x_{0}\right)\right),\hat{F}\left(x_{0}\right)+z_{\alpha /2}\sigma \left(\hat{F}\left(x_{0}\right)\right)], \end{array}$$
  where *z*_*α*/2_ = Φ^−1^(1 − *α*/2), with Φ(⋅) the standard Gaussian CDF. This interval is easy to compute, but there are several assumptions. In a practical situation $\sigma (\hat{F}\left(x_{0}\right))$ is estimated using a bootstrap estimator of the standard error of $\hat{F}\left(x_{0}\right)$. Drawing *B* bootstrap resamples, $X_{1}^{*j},X_{2}^{*j},\ldots ,X_{n}^{*j}$, *j* = 1, 2, …, *B*, from the original sample, *X*_1_, *X*_2_, …, *X*_*n*_, and computing the bootstrap (parametric or nonparametric) estimators, ${\hat{F}}^{*j}\left(x_{0}\right)$, *j* = 1, 2, …, *B*, then $\sigma (\hat{F}\left(x_{0}\right))$ is estimated by the standard deviation of the replicates, i.e., by:
  $$\begin{array}{c} \hat{\sigma }\left({\hat{F}}^{*}\left(x_{0}\right)\right)=\sqrt{\frac{1}{B-1}\sum_{j=1}^{B}\left({\hat{F}}^{*j}\left(x_{0}\right)-{\bar{\hat{F}}}^{*}\left(x_{0}\right)\right)}, \end{array}$$
  where ${\bar{\hat{F}}}^{*}\left(x_{0}\right)=\frac{1}{B}\sum_{j=1}^{B}{\hat{F}}^{*j}\left(x_{0}\right)$.
**The percentile bootstrap CI**. Given an initial confidence level, 1 − *α*, and a point of the CDF domain, *x*_0_, we start by approximating the sampling distribution of $\hat{F}\left(x_{0}\right)$. This is done using its bootstrap distribution, from which it is easy to obtain a pointwise (1 − *α*)-CI, estimating the corresponding quantiles. The process is the following:
  1. Draw *B* bootstrap resamples, $X_{1}^{*j},X_{2}^{*j},\ldots ,X_{n}^{*j}$, *j* = 1, 2, …, *B*, from the original sample, *X*_1_, *X*_2_, …, *X*_*n*_.
  2. Compute the bootstrap (parametric or nonparametric) estimators, ${\hat{F}}^{*j}\left(x_{0}\right)$, *j* = 1, 2, …, *B*, using the bootstrap resamples.
  3. Now, the $\frac{\alpha }{2}$ and $1-\frac{\alpha }{2}$ quantiles of the bootstrap distribution are computed: ${\hat{F}}^{*(\left\lceil \frac{\alpha }{2}B\right\rceil )}\left(x_{0}\right)$ and ${\hat{F}}^{*(\left\lceil \left(1-\frac{\alpha }{2}\right)B\right\rceil )}\left(x_{0}\right)$ (where ⌈*x*⌉ denotes the integer part of *x*).
  4. The final (1 − *α*) (percentile) bootstrap CI for *F*(*x*_0_) is
    $$\begin{array}{c} {}[{\hat{F}}^{*(\left\lceil \frac{\alpha }{2}B\right\rceil )}\left(x_{0}\right),{\hat{F}}^{*(\left\lceil \left(1-\frac{\alpha }{2}\right)B\right\rceil )}\left(x_{0}\right)]. \end{array}$$
**The basic bootstrap CI**. The basic bootstrap CI transforms the distribution of the replicates by subtracting the observed statistic. The quantiles of the transformed sample are used to determine the confidence limits. The algorithm is the following:
  1. Obtain a bootstrap sample, $X_{1}^{*j},X_{2}^{*j},\ldots ,X_{n}^{*j}$, from the original sample, *X*_1_, *X*_2_, …, *X*_*n*_.
  2. With this bootstrap sample, compute an approximate bootstrap version of $D_{n}\left(x_{0}\right)=\hat{F}\left(x_{0}\right)-F\left(x_{0}\right)$, using
    $$\begin{array}{c} D_{n}^{*}\left(x_{0}\right)={\hat{F}}^{*}\left(x_{0}\right)-\tilde{F}\left(x_{0}\right), \end{array}$$ (7)
    where $\tilde{F}\left(x_{0}\right)$ is a pilot (parametric or nonparametric) estimator of *F*(*x*_0_).
  3. Repeat steps 1 and 2 a large number of times *B*. Therefore, after this step, *B* values of the bootstrap distribution $D_{n}^{*}\left(x_{0}\right)$ are avaliable.
  4. Compute the $\frac{\alpha }{2}$ and $1-\frac{\alpha }{2}$ quantiles of the bootstrap distribution: $D_{n}^{*(\left\lceil \frac{\alpha }{2}B\right\rceil )}\left(x_{0}\right)$ and $D_{n}^{*(\left\lceil \left(1-\frac{\alpha }{2}\right)B\right\rceil )}\left(x_{0}\right)$, and the corresponding percentile CI for $D_{n}^{*}\left(x_{0}\right)$:
    $$\begin{array}{c} D_{n}^{*(\left\lceil \frac{\alpha }{2}B\right\rceil )}\left(x_{0}\right)\leq \hat{F}\left(x_{0}\right)-F\left(x_{0}\right)\leq D_{n}^{*(\left\lceil \left(1-\frac{\alpha }{2}\right)B\right\rceil )}\left(x_{0}\right). \end{array}$$
  5. The final bootstrap confidence interval for *F*(*x*_0_) is
    $$\begin{array}{c} \left[\hat{F}\left(x_{0}\right)-D_{n}^{*(\left\lceil \left(1-\frac{\alpha }{2}\right)B\right\rceil )}\left(x_{0}\right),\hat{F}\left(x_{0}\right)-D_{n}^{*(\left\lceil \frac{\alpha }{2}B\right\rceil )}\left(x_{0}\right)\right] \end{array}$$
    or, considering the expression Eq (7), equivalently,
    $$\begin{array}{c} {}[\hat{F}\left(x_{0}\right)+\tilde{F}\left(x_{0}\right)-{\hat{F}}^{*(\left\lceil \left(1-\frac{\alpha }{2}\right)B\right\rceil )}\left(x_{0}\right),\hat{F}\left(x_{0}\right)+\tilde{F}\left(x_{0}\right)-{\hat{F}}^{*(\left\lceil \frac{\alpha }{2}B\right\rceil )}\left(x_{0}\right)], \end{array}$$
    where ${\hat{F}}^{*(\left\lceil \frac{\alpha }{2}B\right\rceil )}\left(x_{0}\right)$ and ${\hat{F}}^{*(\left\lceil \left(1-\frac{\alpha }{2}\right)B\right\rceil )}\left(x_{0}\right)$ are the corresponding $\frac{\alpha }{2}$ and $1-\frac{\alpha }{2}$ quantiles of the bootstrap distribution ${\hat{F}}^{*}\left(x_{0}\right)$.
**The Bias Corrected and accelerated (BCa) CI**. Some modifications of the previous methods have been proposed to get CIs with better theoretical properties and better performance in practice. The BCa approach is one of these techniques. The CIs constructed with this procedure are a modified version of the percentile CIs. They are second-order accurate and transforming respecting.
  Drawing *B* bootstrap resamples, $X_{1}^{*j},X_{2}^{*j},\ldots ,X_{n}^{*j}$, *j* = 1, 2, …, *B*, from the original sample, *X*_1_, *X*_2_, …, *X*_*n*_, and computing the bootstrap (parametric or nonparametric) estimators ${\hat{F}}^{*j}\left(x_{0}\right)$, *j* = 1, 2, …, *B*, the (1 − *α*) BCa CI is
  $$\begin{array}{c} {}[{\hat{F}}^{*(\left\lceil \alpha_{1}B\right\rceil )}\left(x_{0}\right),{\hat{F}}^{*(\left\lceil \alpha_{2}B\right\rceil )}\left(x_{0}\right)], \end{array}$$
  where
  $$\begin{array}{c} \alpha_{1}=\Phi \left({\hat{z}}_{0}+\frac{{\hat{z}}_{0}+z_{\alpha /2}}{1-\hat{a}\left({\hat{z}}_{0}+z_{\alpha /2}\right)}\right) \end{array}$$
  and
  $$\begin{array}{c} \alpha_{2}=\Phi \left({\hat{z}}_{0}+\frac{{\hat{z}}_{0}+z_{1-\alpha /2}}{1-\hat{a}\left({\hat{z}}_{0}+z_{1-\alpha /2}\right)}\right), \end{array}$$
  with *z*_*α*_ = Φ^−1^(*α*), and ${\hat{z}}_{0}$ and $\hat{a}$ estimators of the bias correction and the acceleration adjustment, respectively. The bias correction factor is in effect measuring the median bias of the replicates ${\hat{F}}^{*}\left(x_{0}\right)$ for $\hat{F}\left(x_{0}\right)$. It is estimated by:
  $$\begin{array}{c} {\hat{z}}_{0}=\Phi^{-1}\left(\frac{1}{B}\sum_{j=1}^{B}I\left({\hat{F}}^{*j}\left(x_{0}\right)<\hat{F}\left(x_{0}\right)\right)\right), \end{array}$$
  where *I*(⋅) is the indicator function. Note that ${\hat{z}}_{0}=0$ if $\hat{F}\left(x_{0}\right)$ is the median of the bootstrap replicates. The acceleration constant refers to the rate of change of the standard error of $\hat{F}\left(x_{0}\right)$ with respect to the actual value of the CDF. It can be estimated in various ways [20], for instance, from the equation
  $$\begin{array}{c} \hat{a}=\frac{\sum_{i=1}^{n}{\left(\bar{\hat{F}{\left(x_{0}\right)}_{\left(\cdot \right)}}-\hat{F}{\left(x_{0}\right)}_{\left(i\right)}\right)}^{3}}{6{\left[\sum_{i=1}^{n}{\left(\bar{\hat{F}{\left(x_{0}\right)}_{\left(\cdot \right)}}-\hat{F}{\left(x_{0}\right)}_{\left(i\right)}\right)}^{2}\right]}^{3/2}}, \end{array}$$
  where $\hat{F}{\left(x_{0}\right)}_{\left(i\right)}$ denotes the *i*-th jackknife nonparametric estimate of the CDF, and $\bar{\hat{F}{\left(x_{0}\right)}_{\left(\cdot \right)}}$ is the arithmetic mean of all jackknife estimates.

#### Simultaneous confidence intervals

In the previous section, different bootstrap approaches to construct CIs for the CDF, *F*, evaluated in a specific point, *x*_0_, were described. However, in many cases, the target is not the estimation of the single value *F*(*x*_0_), but the estimation of the CDF, *F*. In these cases, the development of reliable methods to construct simultaneous CIs, i.e., confidence bands, are of great interest. A first approximation to construct a 1 − *α* confidence band could be simply computing the 1 − *α* pointwise CIs in a grid of *k* selected points using one of the previous techniques, and considering this set of pointwise CIs as the corresponding band. No matter the method used to produce the individuals pointwise CIs, the band obtained with this procedure can be named as *uncorrected*, because although individual confidence intervals have approximately the nominal coverage probability (1 − *α*) when they are considered separately (for a particular grid point), the probability that the whole curve is included in the band depicted by the whole set of intervals is much smaller. This is known as the multiple range testing problem [37] or the false discovery rate in high dimensional statistical problems [38].

A classical way to correct for multiple testing and to get multiple level which is much closer to the desired *α* is the popular Bonferroni approach [25]. In a hypothesis testing context, the idea behind this approach, when *k* hypotheses are simultaneously tested, is to consider a new significance level, *α*_*Bonf*_ = *α*/*k*, and compute *k* individual tests using this new level. The resulting multiple test has a multiple level which is much closer to the desired *α*. Obviously, once the Bonferroni significance level is calculated, the corresponding CIs could be obtained using any of the methods described in the previous section. For simplicity, just the basic and the BCa techniques are used in the experiments. In general, no matter the method used, the generic term *Bonferroni band* is used here.

However, the Bonferroni approach is usually a conservative procedure. In this context, this means that the joint coverage probability of the confidence band would be larger than the desired 1 − *α*. Trying to solve this problem, an iterative method is proposed. Starting from the conservative Bonferroni approach and the anticonservative individual testing approach (only the basic method are used in these experiments), the following algorithm finds an approximate (1 − *α*)-confidence interval, with a given approximation error *δ* (typically *δ* is small in comparison with the nominal *α*, for instance $\delta =\frac{\alpha }{10}$):

1. Fix $\alpha_{\text{low}}^{\left(0\right)}=\alpha_{\text{Bonf}}=\frac{\alpha }{k}$ and $\alpha_{\text{high}}^{\left(0\right)}=\alpha$. Fix the iteration number, *i* = 0.
2. Compute $\alpha_{\text{mean}}^{\left(i\right)}=\frac{\alpha_{\text{low}}^{\left(i\right)}+\alpha_{\text{high}}^{\left(i\right)}}{2}$.
3. Use the bootstrap resamples to compute individual confidence intervals with $1-\alpha_{\text{low}}^{\left(i\right)}$, $1-\alpha_{\text{mean}}^{\left(i\right)}$ and $1-\alpha_{\text{high}}^{\left(i\right)}$ confidence levels.
4. Compute, with the same bootstrap resamples, the proportion of bootstrap curves that are included in each of these confidence bands. These proportions satisfy $p_{\text{low}}^{\left(i\right)}\geq p_{\text{mean}}^{\left(i\right)}\geq p_{\text{high}}^{\left(i\right)}$, $p_{\text{low}}^{\left(i\right)}\geq 1-\alpha \geq p_{\text{high}}^{\left(i\right)}$ and $p_{\text{low}}^{\left(i\right)}>p_{\text{high}}^{\left(i\right)}$.
5. If $p_{\text{mean}}^{\left(i\right)}\geq 1-\alpha$, then define $\alpha_{\text{low}}^{\left(i+1\right)}=\alpha_{\text{mean}}^{\left(i\right)}$ and $\alpha_{\text{high}}^{\left(i+1\right)}=\alpha_{\text{high}}^{\left(i\right)}$. Otherwise define $\alpha_{\text{low}}^{\left(i+1\right)}=\alpha_{\text{low}}^{\left(i\right)}$ and $\alpha_{\text{high}}^{\left(i+1\right)}=\alpha_{\text{mean}}^{\left(i\right)}$.
6. Stop at step *i* if $|p_{\text{mean}}^{\left(i\right)}-\left(1-\alpha \right)|<\delta$. Otherwise increase *i* in one unit and repeat Steps 2–5.

The final approximate (1 − *α*) simultaneous CIs are those obtained for level $1-\alpha_{\text{mean}}^{\left(i\right)}$ in the last iteration. The band produced is denoted by the terms *corrected band*.

This procedure to correct the Bonferroni method follows different ideas to other methods proposed in the literature for this purpose (see [39], where five modified Bonferroni procedures are compared). The technique used here takes advantage of the bootstrap resamples obtained to construct the CIs to approximate the correct confidence level. This gives a simple and very fast general method, correcting (at least partially) the error made if the individual CIs (computed using BCa or basic bootstrap) were used as simultaneous CIs. The iterative process to correct the confidence level follows the same principles as those used in the very simple bisection method to approximate a solution of a non-linear equation. The parameter *δ* represents the error that the user is willing to assume (it was checked that the suggestion of *δ* = *α*/10 provides fast and good results). This approach produce CIs with levels in the continuum between those used to construct uncorrected or Bonferroni CIs, being more or less closer to each one of them depending on the number, *k*, of grid points.

## Simulation study

In this section, the pointwise and simultaneous bootstrap CIs described in the previous section are compared, via a simulation study, using synthetic data. Specific code to carry out the numerical analysis shown in this section and the following (focus on real data applications) was developed using the free statistical software R [40]. In these programs, some existing R libraries (cited below) to generate and fit the different parametric and nonparametric models used in our research were also employed. Initially, the main interest is the CDF. From Eqs (1)–(3), it is clear that once the CDF is estimated, other important functions can be immediately approximated, with the goodness of these estimates being directly determined by the quality of CDF estimation. To check this fact empirically, in the second part of the study, the same kind of analysis for the return level function is repeated.

Starting with the analysis of the CDF and using a parametric methodology, the Gamma, Log-normal, Gumbel and Weibull distributions (all of them with two unknown parameters) were considered as parametric families to which the unknown CDF could belong. These parametric distributions are traditionally used to fit annual instantaneous peak discharges of a river [41]. Without loss of generality, this study could be performed for any other distribution. The parametric models were fitted and the corresponding unknown parameters estimated using the R library nsRFA [42]. Regarding the nonparametric approach, the kernel estimator given in Eq (6) was used, with a smoothing parameter selected by the plug-in method described in [31]. The R library Kerdiest [33] was used to fit the nonparametric estimator. On the other hand, in the first part of the study, the Gamma distribution was considered as the parent distribution, generating the synthetic data from this variable. This is a distribution commonly employed in this context. Specifically, a Gamma distribution with probability density function $f\left(x\right)=\frac{1}{\theta^{k}\Gamma \left(k\right)}x^{k-1}e^{-\frac{x}{\theta }}$, with shape parameter *k* = 10 and scale parameter *θ* = 2.6, was used. The values of these parameters were chosen consistently with those estimated from the discharge data of the next section [10].

In the simulation experiments, CIs for the CDF evaluated at specific quantiles, corresponding to more or less extreme values of the variable, were computed. Observing Eq (3), it is easy to see that return-level estimation is equivalent to quantile estimation. Taking this into account, the procedure employed has the following steps. First, a sample of size *n* from a Gamma distribution, *X*_1_, *X*_2_, …, *X*_*n*_, is generated. Second, a grid of *j* particular periods of time (years) *T* = *T*_1_, *T*_2_, …, *T*_*j*_ are considered, and the values 1 − (1/*T*) calculated. Then, using the sample data, the *j* sample quantiles corresponding to the probabilities $1-\frac{1}{T_{1}},\ldots ,1-\frac{1}{T_{j}}$ are computed. Denoting these quantiles by *q*_1_, …, *q*_*j*_, the values *F*(*q*_*j*_) are estimated by means of $\hat{F}\left(q_{j}\right)$, where $\hat{F}\left(\cdot \right)$ can be a parametric or a nonparametric estimator. Finally, the different CIs are constructed.

Three different sets of *T* values (denoted by *T*^(1)^, *T*^(2)^ and *T*^(3)^, respectively) were considered, obtaining thus the corresponding different sets of return periods. The periods of time (in years) in these sets are, respectively, *T*^(1)^ = {4, 5, 6, 7, 8, 20, 50, 100, 200}, *T*^(2)^ = {2, 4, 5, 10, 20, 25, 50, 100, 200} and *T*^(3)^ = {4, 6, 8, 10, 12, 14, 16, 18, 20}. The different values of *T* allow to control the number of extreme values where the CDF is estimated. For example, the last values for *T* in *T*^(1)^ and *T*^(2)^ are 50,100 and 200. These high return-periods correspond to extreme quantiles of probabilities 0.98,0.99 and 0.995, respectively. Nevertheless, the set of values included in *T*^(3)^ produce not so large quantiles. In this way, the performance of the different methods to construct CIs in different situations can be checked.

Different sample sizes, *n*, have been considered, obtaining similar conclusions for all of them. For the sake of brevity, only the case of *n* = 100 is presented here. On the other hand, following [24], the number of bootstrap replicas was *B* = 40*n* (i.e., 4,000 for the case of *n* = 100). Each simulated setting was repeated 1,000 times.

For each simulation replica, the *j* bootstrap CIs (in each of the *j* grid points) were computed using the methods standard normal bootstrap, percentile bootstrap, basic bootstrap and BCa. These techniques are designed to obtain pointwise CIs, although they can also be used in a simultaneous way to produce confidence bands. However, and with the aim of producing more accurate simultaneous CIs, the Bonferroni approach was also applied to the CIs obtained by the basic bootstrap and the BCa methods. Moreover, the iterative algorithm indicated in the previous section to correct the Bonferroni technique was also employed. This iterative process was just applied starting from the CIs obtained by the basic method in the simulations, although other alternative methods could also be used. Therefore, seven methods to construct CIs were tested, four of them (standard normal bootstrap, percentile bootstrap, basic bootstrap and BCa methods) designed to obtain pointwise CIs (in what follows, denoted by Stand, Perc, Basic and BCa, respectively), and three based on modifications of some of the previous ones (Bonferroni applied to the basic and BCa intervals, and the iterative process starting from the basic CIs), to be used when the interest is to obtain simultaneous CIs (they are denoted by Bon-Basic, Bon-BCa and Corr-Bon, respectively).

Once the pointwise or simultaneous CIs were computed, the corresponding pointwise and simultaneous coverage probabilities of the CIs constructed with the different procedures were estimated. For this, it was checked if the real value of the CDF at the corresponding grid point is or not inside the interval. After the 1,000 repetitions, the pointwise coverage probabilities can be estimated. Following a similar process, it was also computed the simultaneous coverage probabilities, checking if all the values of the theoretical CDF at the grid points were inside the corresponding intervals.

Table 1 shows the results of these simulations with *α* = 0.05 and *n* = 100. For each model, the left column (denoted with the letter ‘P.’) shows the estimated pointwise coverage percentage (interval coverage), and the right column (denoted with the letter ‘S.’), the simultaneous coverage percentage (band coverage).

**Table 1:** Pointwise (columns denoted with the letter ‘P.’) and simultaneous (columns denoted with the letter ‘S.’) coverage percentage of the different methods used to construct CIs.

		Nonparam.		Gamma		Fit Log-Norm		Gumbel		Weibull	
*T*	method	P.	S.	P.	S.	P.	S.	P.	S.	P.	S.
*T*^(1)^	Stand	83.20	54.10	84.35	77.10	83.70	70.00	78.40	53.90	71.40	32.00
	Perc	88.87	62.50	94.03	89.40	89.81	73.30	79.07	38.90	78.92	41.80
	Basic	86.77	57.90	90.03	78.50	93.81	87.00	87.27	68.10	66.83	12.80
	BCa	89.53	63.70	95.02	91.00	87.25	65.80	74.81	26.90	82.34	51.40
	Bon-Basic	89.54	61.70	94.47	84.80	99.04	98.50	98.65	96.70	74.93	22.10
	Bon-BCa	92.75	72.00	96.96	94.50	91.04	74.30	78.70	34.90	87.32	62.10
	Corr-Bon	89.03	61.10	91.90	81.60	96.28	92.30	91.08	77.80	70.01	16.10
*T*^(2)^	Stand	85.10	52.90	85.90	74.80	86.56	68.80	79.23	52.40	71.40	32.00
	Perc	88.63	59.20	93.85	86.60	89.77	69.60	77.10	35.00	78.40	39.90
	Basic	86.28	54.80	89.16	75.90	93.67	82.80	85.97	64.30	66.65	14.80
	BCa	89.38	59.80	95.14	88.30	85.83	61.70	70.82	22.00	82.60	49.30
	Bon-Basic	88.72	61.10	93.75	84.60	98.95	96.90	98.61	95.70	73.20	25.30
	Bon-BCa	93.21	71.70	97.96	94.60	91.52	73.20	77.92	36.50	89.22	65.20
	Corr-Bon	88.37	60.30	91.68	81.20	97.04	91.10	92.57	79.80	70.20	19.50
*T*^(3)^	Stand	89.94	72.70	85.89	76.30	86.60	72.50	84.10	66.70	82.10	57.80
	Perc	94.92	83.80	94.20	87.90	94.40	84.20	90.50	72.50	89.80	68.00
	Basic	94.13	81.20	92.27	85.20	94.00	85.00	91.40	78.10	85.40	55.90
	BCa	95.04	83.70	95.50	89.70	92.50	77.20	86.60	64.40	91.40	73.90
	Bon-Basic	97.70	91.80	97.03	93.50	98.80	97.10	98.90	97.20	92.80	75.20
	Bon-BCa	98.74	94.90	98.07	95.10	96.50	88.40	91.10	73.00	95.70	86.20
	Corr-Bon	96.94	89.60	94.80	89.50	96.60	91.10	95.20	86.20	88.90	64.80

Data are simulated from a Gamma(10,2.6) distribution, and fitted using a nonparametric fit and parametric fits assuming a Gamma, Log-normal, Gumbel and Weibull distributions. The percentages are rounded using two significant figures.

Some conclusions can be deduced from the results included in Table 1. Ideally, the pointwise methods (Stand, Perc, Basic and BCa) should achieve a pointwise coverage percentage of (or close to) 95% (column ‘P.’), while the simultaneous coverage percentage (column ‘S.’) of the simultaneous procedures (Bon-Basic, Bon-BCa and Corr-Bon) should be close to 95%. On the other hand, due to the multiple range testing, it is expected that the simultaneous coverage of the approaches Stand, Perc, Basic and BCa should be smaller than their nominal value (95%, in this case). Note that the methods Bon-Basic, Bon-BCa and Corr-Bon are designed to try to overcome this issue. Taking these arguments into account, it is clear that the best results for all the approaches were obtained when the set of return-periods *T*^(3)^ was considered. Note that this set of values does not contain large values, while *T*^(1)^ and *T*^(2)^ include the values 50, 100 and 200, producing extreme quantiles, where it is much more difficult to obtain reliable distribution estimations. Additionally, the best performance of the different methods was obtained when the data were fitted with a Gamma distribution. This is quite natural because the data were generated from this kind of variable. Nevertheless, using other parametric fits led to worse results, especially if the shape of the fitted distribution differs from a Gamma variable (a Weibull, for instance). In this sense, the nonparametric approach represents a valid alternative. The results obtained with this methodology were better than those using a parametric model different to the one employed to generate the data (Gamma), but they were still close to the best percentages obtained under a Gamma fit. Therefore, the nonparametric approach appears reasonably robust to model misspecification.

Regarding the different methods, when the aim is to construct pointwise CIs, all the pointwise approaches seem to be, in general, anticonservative. In this framework, the BCa approach (BCa) produced the best results. It can be easily observed that the pointwise coverage percentages of the BCa technique are the closest to their nominal value (95%) among the different pointwise methods. This is specially remarkable in the case of considering *T*^(3)^, using a nonparametric fit or the reference Gamma distribution (with percentages of 95.04% and 95.50%, respectively). The anticonservative nature of the pointwise methods made the simultaneous approaches (Bon-Basic, Bon-BCa and Corr-Bon) to give, in general, better results than the pointwise methods when they are considered pointwisely. However, this could be an artificial effect, consequence of the poor performance of pointwise methods in some scenarios (specially using *T*^(1)^ or *T*^(2)^, and assuming a Gumbel or a Weibull distribution). As for the simultaneous coverage percentages, it is clear that the designed methods for constructing confidence bands worked better than the pointwise approaches. The band coverage (simultaneous) percentages for Bon-Basic, Bon-BCa and Corr-Bon were closer to their nominal values (95%) than those computed with the approaches denoted by Stand, Perc, Basic and BCa, although, in general, all of them tended to be anticonservative. In this simultaneous context, the best performance was achieved by Bon-BCa (Bonferroni applied to BCa intervals), especially when *T*^(3)^ is considered and a nonparametric fit or the reference Gamma distribution was used (band coverage percentages of 94.90% and 95.10%, respectively). The approach Bon-Basic (Bonferroni applied to the basic intervals) gave simultaneous coverage probabilities over and above the nominal value, depending on the scenario; while the method Corr-Bon (designed to correct the conservative feature of the Bonferroni approach) seems to be, in general, anticonservative.

In the second part of the simulation study, the interest were the return levels, defined in Eq (3). A similar process to that previously described for the CDF was performed. The different pointwise and simultaneous CIs were computed, and their corresponding pointwise and simultaneous coverages calculated. The general procedure follows the same steps as before, but with the obvious changes in the estimation process when the target is the return level function instead of the CDF. Here, a heavy-tailed distribution as the parent distribution was used, because this type of models are widely employed in extreme value problems in hydrology. Specifically, a GEV distribution Eq (5), with location parameter *μ* = 1555.73, scale parameter *σ* = 613.57, and shape parameter *γ* = 0.10, was employed to generate the artificial data. This distribution corresponds to the one fitted to the real data set of annual peak instantaneous flows of the Spanish river analyzed in the next section. Note that, in the nonparametric case, return level estimation is obtained by means of a numerical approximation to the root of the equation $p-{\hat{F}}_{h}\left(x_{p}\right)=0$, with 0 < *p* < 1. This process always implies some extra computing time that may be high when, additionally, one has to select a smoothing parameter and bootstrap methods are being used. Also note that the comparisons based on simulations require repeating the whole number of calculations a large number of times (1,000 in this case). Taking this into account, in this part of the study, just the previous scenarios *T*^(2)^ and *T*^(3)^ were considered. Moreover, to fit the data, the nonparametric estimator of the return levels obtained from Eq (6), and the parametric estimator, given in Eq (4), assuming that the data follow a (correct) GEV distribution and also a (misspecified) Gumbel model were considered. Table 2 contains the same type of information as that presented in Table 1, but for the return levels.

**Table 2:** Pointwise (columns denoted with the letter ‘P.’) and simultaneous (columns denoted with the letter ‘S.’) coverage percentage of the different methods used to construct CIs.

		Nonparam.		Fit GEV		Gumbel	
*T*	method	P.	S.	P.	S.	P.	S.
*T*^(2)^	Stand	87.10	52.80	89.46	75.90	81.74	47.60
	Perc	84.46	35.00	93.88	85.80	85.40	44.40
	Basic	80.06	24.10	93.54	83.80	83.23	38.50
	Bca	84.12	33.40	94.20	86.10	83.90	40.90
	Bon-Basic	86.07	33.20	97.84	94.60	95.50	79.90
	Bon-BCa	87.18	39.30	97.01	93.20	89.90	57.90
	Corr-Bon	84.59	30.30	96.48	91.40	88.84	54.60
*T*^(3)^	Stand	88.10	77.30	86.59	78.20	84.52	73.10
	Perc	94.70	88.50	94.27	90.00	94.13	85.90
	Basic	90.60	79.60	94.58	89.70	93.63	84.90
	Bca	94.66	88.40	94.80	90.80	94.34	87.30
	Bon-Basic	97.22	92.70	98.42	97.30	98.57	97.00
	Bon-BCa	97.92	94.40	96.62	94.00	96.10	91.80
	Corr-Bon	94.36	86.40	96.49	93.40	95.20	88.60

Data are simulated from a GEV(1555.73,613.57,0.10) distribution, and fitted using a nonparametric fit and parametric fits assuming a GEV and a Gumbel distributions. The percentages are rounded using two significant figures.

As expected, very similar conclusions to those pointed out in the case of the CDF can be reproduced here for the different procedures used to construct the CIs. However, some differences are observed in the results obtained with the nonparametric estimator, when the interest is to construct simultaneous CIs and the set *T*^(2)^ (containing some high return-periods) is considered. In that case, the modifications of the pointwise procedures (Bon-Basic, Bon-BCa and Corr-Bon) seem to be too much anticonservative, with band coverage percentages far from the ideal 95%. This fact may be due, among other factors, to the error made by the numerical method used to approximate the corresponding quantiles. More efficient techniques could be used for this task, but with a higher computing time. In any case, it can be observed that even assuming a Gumbel distribution (which is not very similar to the parent GEV distribution) to fit the data, this effect is at least partially corrected. Hence, in situations when the parametric model cannot be exactly specified, instead of using a nonparametric approach (as suggested in the CDF case), here, a clear improvement is obtained if a proper parametric model (selected by means of goodness-of-fit tests) is considered to be fitted to the data.

Therefore, to sum up, in the case of the CDF, if the interest is to compute individual CIs, the suggestion is to use the BCa approach (BCa), but if the concern is to compute simultaneous CIs, the pointwise methods are far from achieving the nominal value, while the technique denoted by Bon-BCa (Bonferroni applied to BCa intervals) corrects (at least partially) this bias in a simple and fast way, and this is the recommendation. On the other hand, when it is not feasible to know the distribution of the data under study, the proposal is to use a nonparametric model to estimate the CDF and obtain the corresponding pointwise or simultaneous CIs. In the case of the return levels, the same conclusions hold, except that to construct simultaneous CIs when the distribution of the data is not known, it is suggested to fit a proper parametric model, using a goodness-of-fit test, instead of applying nonparametric approaches as in CDF case.

## Applications

In this section, several data sets of annual maximum peak discharges are used to show the practical application of the different CI estimation procedures. Specifically, four series from the US Geological Survey (USGS), measured in cfs (cubic feet per second), and a data set of a Spanish river (Ebro river), measured in cumecs (cubic meters per second), have been selected.

### US river data

As a first case study, the different methods previously described have been applied to construct pointwise and simultaneous CIs for the CDF to four USGS series extracted from a larger collection studied and described in [41] and in [10]. In [10], the authors applied several goodness-of-fit tests to obtain the best theoretical parametric distribution fitting each series. Here, four particular series fitted by a different parametric model in that work, with identification (USGS notation) 02055000 (Roanoke River, VA), 01463500 (Delaware River, NJ), 03011020 (Allegheny River, NY) and 11152000 (Arroyo River, CA) (see table 2 of [10] for a detailed description), have been chosen. The best distributions to fit these series (selected by means of the Akaike Information Criterion (AIC), [43]) are, respectively, the Gamma, the Log-normal, the Gumbel and the Weibull distributions (see table 3 of [10]).

In this application, the process followed to construct the CIs is similar to that described in the previous simulation section (obviously, now, only one sample data is available for each river). The set of return periods considered here was *T* = {5, 10, 20, 100, 200, 500, 1000}. This means that, applying Eq (3), the corresponding CIs were computed in the quantiles of probabilities 0.8, 0.9, 0.95, 0.99, 0.995, 0.998 and 0.999, respectively. Tables 3–6 show the CIs constructed with the different methods (Stand, Perc, Basic and BCa, designed to obtain pointwise CIs; and Bon-Basic, Bon-BCa and Corr-Bon, designed to obtain simultaneous CIs) for each one of the rivers considered. These tables have two parts. In the top part of each table, the CIs obtained in the seven extreme quantiles when the corresponding parametric model that best fit the data of each river is assumed are presented, while the bottom parts show the CIs constructed using a nonparametric model.

**Table 3:** Lower and upper limits of pointwise (Stand, Perc, Basic and BCa) and simultaneous (Bon-Basic, Bon-BCa and Corr-Bon) CIs for the CDF at quantiles with return periods *T* = {5, 10, 20, 100, 200, 500, 1000}, using a Gamma parametric model (top part) and a nonparametric model (bottom part). Roanoke River (ID = 02055000).

*T* (years)	Stand		Perc		Basic		BCa		Bon-Basic		Bon-BCa		Corr-Bon	
	Low	Up	Low	Up	Low	Up	Low	Up	Low	Up	Low	Up	Low	Up
Parametric fit (Gamma)														
5	0.68	0.82	0.71	0.84	0.70	0.84	0.71	0.84	0.68	0.86	0.70	0.85	0.70	0.84
10	0.79	0.91	0.81	0.92	0.81	0.92	0.81	0.92	0.79	0.94	0.80	0.92	0.80	0.92
20	0.93	0.99	0.94	0.99	0.94	0.99	0.93	0.99	0.94	1.00	0.93	0.99	0.94	1.00
100	0.96	1.00	0.97	1.00	0.97	1.00	0.96	0.99	0.97	1.00	0.96	1.00	0.97	1.00
200	0.98	1.00	0.98	1.00	0.99	1.00	0.98	1.00	0.99	1.00	0.98	1.00	0.99	1.00
500	0.99	1.00	0.99	1.00	0.99	1.00	0.99	1.00	0.99	1.00	0.98	1.00	0.99	1.00
1000	0.99	1.00	0.99	1.00	0.99	1.00	0.99	1.00	0.99	1.00	0.99	1.00	0.99	1.00
Nonparametric fit														
5	0.71	0.89	0.72	0.87	0.72	0.87	0.72	0.87	0.70	0.90	0.70	0.88	0.71	0.89
10	0.81	0.95	0.83	0.94	0.83	0.94	0.83	0.94	0.82	0.97	0.81	0.95	0.82	0.96
20	0.88	0.98	0.90	0.98	0.91	0.99	0.90	0.98	0.90	1.00	0.89	0.99	0.91	1.00
100	0.96	1.00	0.96	1.00	0.97	1.00	0.96	1.00	0.97	1.00	0.94	1.00	0.97	1.00
200	0.97	1.00	0.97	1.00	0.98	1.00	0.96	1.00	0.98	1.00	0.96	1.00	0.98	1.00
500	0.97	1.00	0.97	1.00	0.98	1.00	0.95	1.00	0.98	1.00	0.95	1.00	0.98	1.00
1000	0.97	1.00	0.98	1.00	0.98	1.00	0.95	1.00	0.98	1.00	0.95	1.00	0.98	1.00

Numbers are rounded using two significant figures.

**Table 4:** Lower and upper limits of pointwise (Stand, Perc, Basic and BCa) and simultaneous (Bon-Basic, Bon-BCa and Corr-Bon) CIs for the CDF at quantiles with return periods *T* = {5, 10, 20, 100, 200, 500, 1000}, using a Log-normal parametric model (top part) and a nonparametric model (bottom part). Delaware River (ID = 01463500).

*T* (years)	Stand		Perc		Basic		BCa		Bon-Basic		Bon-BCa		Corr-Bon	
	Low	Up	Low	Up	Low	Up	Low	Up	Low	Up	Low	Up	Low	Up
Parametric fit (Log-normal)														
5	0.79	0.94	0.71	0.85	0.71	0.85	0.71	0.85	0.69	0.87	0.70	0.86	0.70	0.85
10	0.87	1.00	0.80	0.92	0.80	0.92	0.80	0.92	0.79	0.94	0.80	0.92	0.80	0.93
20	0.96	1.00	0.94	0.99	0.95	1.00	0.93	0.99	0.94	1.00	0.93	0.99	0.95	1.00
100	0.99	1.00	0.99	1.00	0.99	1.00	0.99	1.00	0.99	1.00	0.98	1.00	0.99	1.00
200	0.99	1.00	0.99	1.00	0.99	1.00	0.99	1.00	0.99	1.00	0.99	1.00	0.99	1.00
500	1.00	1.00	0.99	1.00	1.00	1.00	0.99	1.00	1.00	1.00	0.99	1.00	1.00	1.00
1000	1.00	1.00	0.99	1.00	1.00	1.00	0.99	1.00	1.00	1.00	0.99	1.00	1.00	1.00
Nonparametric fit														
5	0.78	0.94	0.73	0.86	0.73	0.87	0.73	0.87	0.71	0.90	0.72	0.88	0.72	0.88
10	0.89	1.00	0.83	0.94	0.84	0.95	0.83	0.94	0.82	0.97	0.82	0.95	0.83	0.96
20	0.94	1.00	0.90	0.98	0.91	0.99	0.90	0.98	0.90	1.00	0.89	0.99	0.90	1.00
100	0.97	1.00	0.96	1.00	0.97	1.00	0.95	1.00	0.97	1.00	0.95	1.00	0.97	1.00
200	0.98	1.00	0.97	1.00	0.98	1.00	0.95	1.00	0.98	1.00	0.94	1.00	0.98	1.00
500	0.98	1.00	0.98	1.00	0.99	1.00	0.96	1.00	0.99	1.00	0.95	1.00	0.99	1.00
1000	0.99	1.00	0.98	1.00	0.99	1.00	0.97	1.00	0.99	1.00	0.96	1.00	0.99	1.00

Numbers are rounded using two significant figures.

**Table 5:** Lower and upper limits of pointwise (Stand, Perc, Basic and BCa) and simultaneous (Bon-Basic, Bon-BCa and Corr-Bon) CIs for the CDF at quantiles with return periods *T* = {5, 10, 20, 100, 200, 500, 1000}, using a Gumbel parametric model (top part) and a nonparametric model (bottom part). Allegheny River (ID = 03011020).

*T* (years)	Stand		Perc		Basic		BCa		Bon-Basic		Bon-BCa		Corr-Bon	
	Low	Up	Low	Up	Low	Up	Low	Up	Low	Up	Low	Up	Low	Up
Parametric fit (Gumbel)														
5	0.73	0.86	0.75	0.88	0.76	0.89	0.75	0.88	0.73	0.92	0.75	0.88	0.75	0.89
10	0.86	0.95	0.87	0.95	0.88	0.96	0.87	0.95	0.87	0.99	0.86	0.95	0.88	0.96
20	0.90	0.97	0.91	0.97	0.92	0.98	0.90	0.97	0.91	1.00	0.90	0.97	0.91	0.98
100	0.97	1.00	0.97	1.00	0.98	1.00	0.97	0.99	0.98	1.00	0.97	0.99	0.98	1.00
200	0.99	1.00	0.99	1.00	1.00	1.00	0.99	1.00	1.00	1.00	0.99	1.00	1.00	1.00
500	1.00	1.00	1.00	1.00	1.00	1.00	1.00	1.00	1.00	1.00	1.00	1.00	1.00	1.00
1000	1.00	1.00	1.00	1.00	1.00	1.00	1.00	1.00	1.00	1.00	1.00	1.00	1.00	1.00
Nonparametric fit														
5	0.68	0.85	0.72	0.86	0.73	0.87	0.72	0.86	0.70	0.90	0.71	0.88	0.72	0.88
10	0.87	1.00	0.85	0.95	0.85	0.95	0.84	0.95	0.83	0.98	0.83	0.96	0.84	0.97
20	0.92	1.00	0.90	0.98	0.91	0.99	0.89	0.98	0.90	1.00	0.88	0.98	0.90	1.00
100	0.96	1.00	0.96	1.00	0.97	1.00	0.96	1.00	0.97	1.00	0.95	1.00	0.97	1.00
200	0.96	1.00	0.97	1.00	0.98	1.00	0.95	1.00	0.98	1.00	0.94	1.00	0.98	1.00
500	0.96	1.00	0.97	1.00	0.98	1.00	0.94	1.00	0.98	1.00	0.94	1.00	0.98	1.00
1000	0.96	1.00	0.97	1.00	0.98	1.00	0.95	1.00	0.98	1.00	0.95	1.00	0.98	1.00

Numbers are rounded using two significant figures.

**Table 6:** Lower and upper limits of pointwise (Stand, Perc, Basic and BCa) and simultaneous (Bon-Basic, Bon-BCa and Corr-Bon) CIs for the CDF at quantiles with return periods *T* = {5, 10, 20, 100, 200, 500, 1000}, using a Weibull parametric model (top part) and a nonparametric model (bottom part). Arroyo River (ID = 11152000).

*T* (years)	Stand		Perc		Basic		BCa		Bon-Basic		Bon-BCa		Corr-Bon	
	Low	Up	Low	Up	Low	Up	Low	Up	Low	Up	Low	Up	Low	Up
Parametric fit (Weibull)														
5	0.75	0.87	0.76	0.87	0.76	0.87	0.76	0.87	0.74	0.90	0.75	0.88	0.76	0.88
10	0.87	0.95	0.88	0.95	0.88	0.95	0.87	0.95	0.87	0.97	0.87	0.95	0.87	0.96
20	0.92	0.98	0.93	0.98	0.93	0.98	0.92	0.98	0.92	0.99	0.92	0.98	0.93	0.99
100	0.96	0.99	0.96	0.99	0.97	1.00	0.96	0.99	0.96	1.00	0.96	0.99	0.97	1.00
200	0.96	1.00	0.96	0.99	0.97	1.00	0.96	0.99	0.96	1.00	0.96	0.99	0.97	1.00
500	0.96	1.00	0.96	0.99	0.97	1.00	0.96	0.99	0.97	1.00	0.96	0.99	0.97	1.00
1000	0.96	1.00	0.96	0.99	0.97	1.00	0.96	0.99	0.97	1.00	0.96	0.99	0.97	1.00
Nonparametric fit														
5	0.71	0.88	0.73	0.87	0.73	0.87	0.73	0.87	0.71	0.9	0.71	0.88	0.72	0.89
10	0.82	0.96	0.84	0.95	0.85	0.95	0.84	0.95	0.83	0.98	0.83	0.96	0.84	0.97
20	0.90	1.00	0.90	0.98	0.91	0.99	0.89	0.98	0.90	1.00	0.88	0.98	0.90	1.00
100	0.96	1.00	0.96	1.00	0.96	1.00	0.95	1.00	0.96	1.00	0.95	1.00	0.96	1.00
200	0.97	1.00	0.97	1.00	0.97	1.00	0.96	1.00	0.97	1.00	0.96	1.00	0.97	1.00
500	0.97	1.00	0.97	1.00	0.97	1.00	0.97	1.00	0.97	1.00	0.96	1.00	0.97	1.00
1000	0.97	1.00	0.97	1.00	0.98	1.00	0.97	1.00	0.97	1.00	0.96	1.00	0.97	1.00

Numbers are rounded using two significant figures.

It can be observed in Tables 3–6 that the CIs computed in the different quantiles contain the corresponding theoretical probabilities, and this happens not only for the parametric models (where the best parametric fits were used), but also considering nonparametric estimations. In general, the parametric CIs are narrower than the nonparametric CIs, especially in the largest quantiles. This is a known drawback of nonparametric estimation when very extreme quantiles are estimated using global bandwidths (as in this paper). This problem could be partially avoided with the use of local bandwidths. Obviously, this approximation would require an extra computing time and, for the sake of brevity, it is not included in the present research. A good feature of the nonparametric CIs is that, in the lower quantiles, their lower and upper limits are very similar to those obtained with the best parametric fits. This is an interesting point if one was interested in performing a similar study with a new data set. When using a parametric approach in practice, a usual first step is model selection. This means to choose the parametric model (e.g., Gamma, Log-normal, Weibull, etc.) that better fits the data, within a reasonable class of models. The model selected may change from one setup to the other. Nonparametric methods do not need of such a “model selection” step, since they are automatically adapted to the data generation process.

As for the different methods to construct CIs, in general, the results obtained with real data sets follow the same lines to those described in the previous simulation section.

From a hydrological point of view, although a general checking of Tables 3–6 could give the impression that the results obtained for the different approaches are very similar, some interesting conclusions can still be deduced. First, in Tables 4 and 5, corresponding to Delaware and Allegheny rivers, respectively, it is observed that, for large values of *T* (mainly 500 and 1000 years) and using the corresponding parametric fits, the upper and lower limits of some intervals are equal to one. This would mean that the current maximum flow level could not be exceeded in future for those values of *T*. This is quite unlikely from a real point of view. These kind of results never appear in the case of CIs constructed using nonparametric fits. From this perspective, nonparametric fits adapt better to the data and can avoid some of these undesirable effects derived from the use of parametric fits. On the other hand, similarly to the results obtained in the simulation study, in general, shorter CIs are constructed using the BCa and Bon-BCa approaches and parametric fits. This means that given a confidence level, these CIs would be more precise. Practitioners can use this result to know, with a larger precision, the time (in years) necessary to reach the possible larger values for the maximum flow of the corresponding rivers. In any case, although a larger precision could be obtained using a parametric fit, the advise is to perform also a nonparametric study, because it is observed that this can help to filter and refine the final conclusions in a practical study.

### A Spanish river case

**Fig 1:**
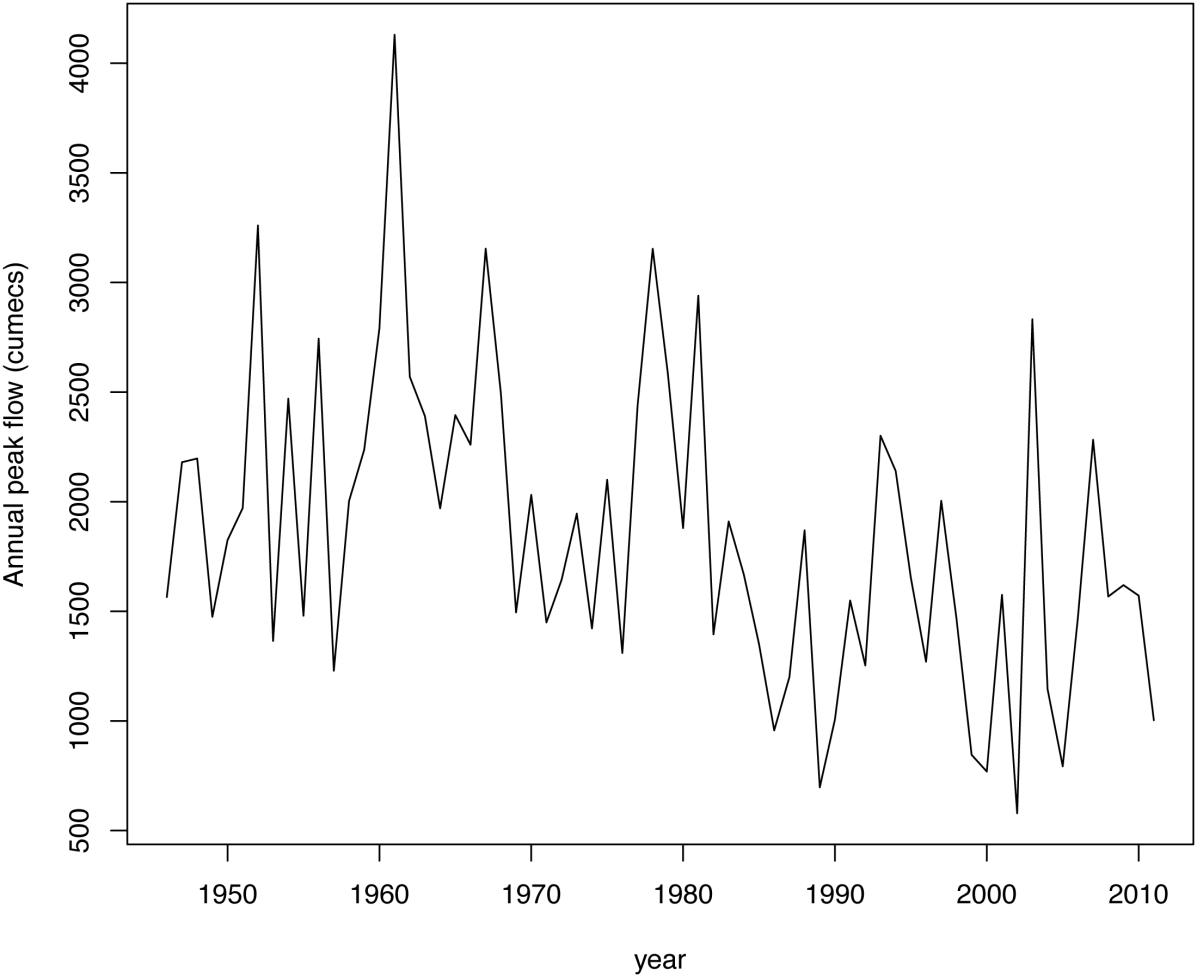
Time series of annual peak flows of Ebro river. Time series of annual peak flows (measured in cumecs) of Ebro river (gaging station at Zaragoza city, Spain).

As a second case study, a similar analysis to that presented in the previous section for the USGS series, but now using a data set from a Spanish river, was performed. The series analyzed consisted of 66 annual peak instantaneous flows (measured in cumecs) corresponding to the Ebro river (gaging station at Zaragoza city), recorded from 1946 to 2001 (included). This series is shown in Fig 1, while Table 7 presents some descriptive values of this series.

**Table 7:** Descriptive study of the 66 annual peak instantaneous flows (measured in cumecs) corresponding to the Ebro river (gaging station at Zaragoza city, Spain).

Min.	1st Quartile	Median	Mean	3rd Quartile	Max.
578.8	1402	1746	1853	2277	4130

This data set has been obtained from the web page of the Ebro Hydrographic Confederation (Spanish Ministry of Agriculture, Food and Environment). The size of this series is one of the largest that can be obtained for the study of annual maximum flows in a Spanish river, because gradual recording and data file storage from rivers was held in Spain after the Second World War.

**Fig 2:**
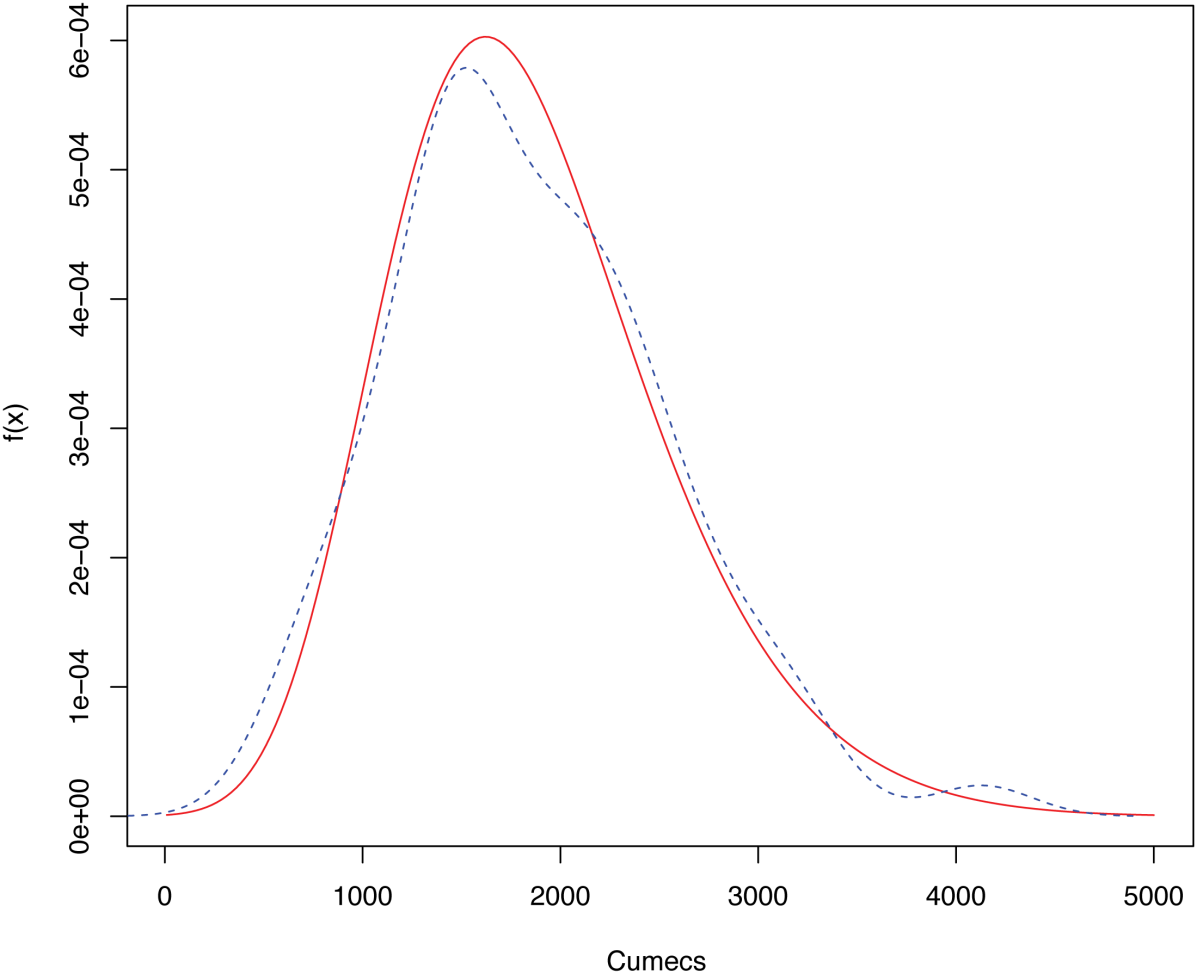
Density estimates of the annual peak instantaneous flows of Ebro river. Density estimates of the annual peak instantaneous flows (measured in cumecs), corresponding to the Ebro river (gaging station at Zaragoza city, Spain). The red solid line represents the parametric density estimate assuming a GEV distribution, the blue dashed line the corresponding nonparametric estimate.

Before proceeding with the comparison of the CIs constructed with the different proposals, an exploratory analysis of the data under consideration was performed and, in order to identify significant data features, parametric and nonparametric density estimates were computed. Specific details of this analysis are given in the Supporting Information (S1 Text). The parametric and nonparametric density estimates of these data are included in Fig 2. The red solid line represents the parametric density estimate assuming a GEV distribution with the parameters indicated above, while the blue dashed line the corresponding nonparametric estimate.

Fig 2 reveals some interesting points. First, the good fit of the GEV distribution to these data set can be confirmed, with the parametric and nonparametric absolute mode estimates of the data being very close. Moreover, the nonparametric estimate presents a relative mode at the right of the figure (extreme values). This fact could be a signal that the fit performed with a GEV variable, perhaps, could be improved using another distribution with heavier tails. This effect could also be an overestimation of the nonparametric estimate (the well-known boundary effect, see e.g. [29]). In this case, the small sample size is a serious drawback for correctly checking the differences between the parametric and nonparametric approaches. However, this plot gives an example on how nonparametric estimation could reveal singularities that the parametric estimation is unable to detect.

**Fig 3:**
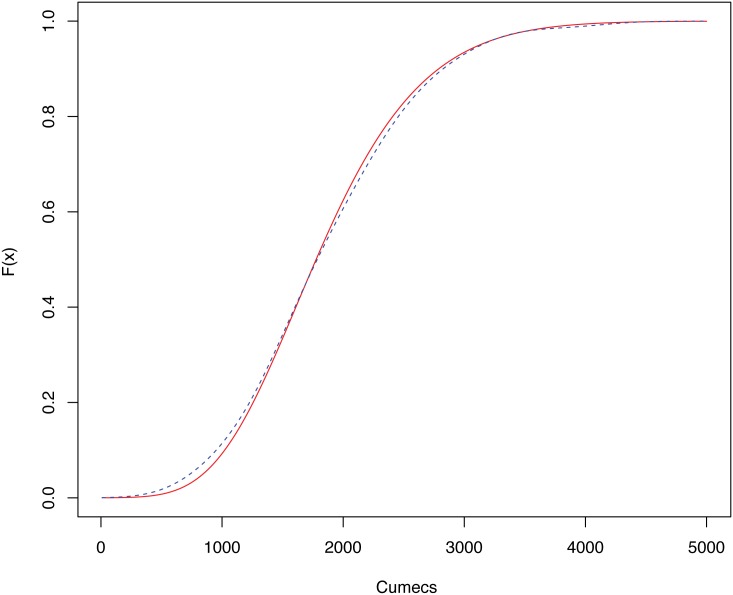
CDF estimates of the annual peak instantaneous flows of Ebro river. CDF estimates of the annual peak instantaneous flows (measured in cumecs), corresponding to the Ebro river (gaging station at Zaragoza city, Spain). The red solid line represents the parametric density estimate assuming a GEV distribution, the blue dashed line the corresponding nonparametric estimate.

**Fig 4:**
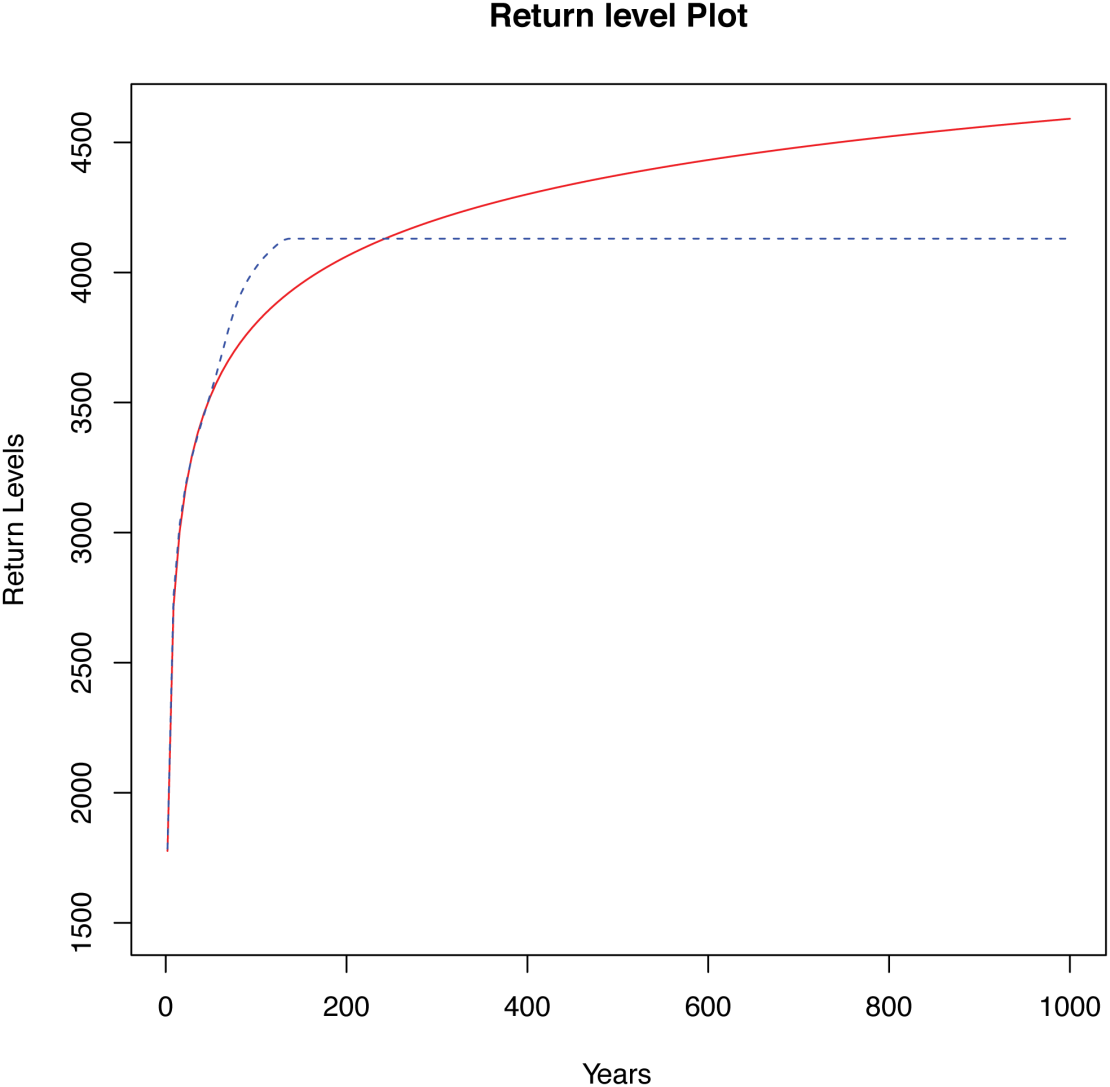
Return level function estimates for different periods of time of Ebro river. Return level function estimates for different periods of time, corresponding to the Ebro river (gaging station at Zaragoza city, Spain). The red solid line represents the parametric density estimate assuming a GEV distribution, the blue dashed line the corresponding nonparametric estimate.

In the case of the USGS series, only the different CIs constructed for the CDF were compared. Now, the case of the return level function was also considered. To carry out this analysis, in a first step, the CDF and the return level function were estimated using parametric and nonparametric methodologies. These estimations are subsequently needed to obtain the different pointwise and simultaneous CIs for these two functions. The nonparametric estimation of the CDF and the return level function was performed using the R package Kerdiest [33]. In both cases, the plug-in bandwidth of Polansky and Baker [31] was used. Fig 3 contains the plots of the CDF estimates, and Fig 4 the corresponding return level function estimates.

The shape of the CDF estimates shown in Fig 3 is very similar. Specific differences can be only observed with a careful examination of small intervals and particular points of interest. Conversely, in the case of the return level estimates (Fig 4), important dissimilitudes can be observed in the curves obtained with the parametric and nonparametric models. The differences in the high values correspond to the above remarked discrepancies observed in Fig 2. In this example, it is important to note that the small sample size of the series can severely affect the obtained nonparametric return level estimations, as indicated in the previous section, by a numerical approximation. This effect is avoided by the parametric techniques, using a direct form obtained through the distribution function to estimate the return levels, in the case of the GEV variable. In this sense, if nonparametric estimates were used to predict the maximum flows, it would turn out that extreme levels would be reached within a shorter period of time than that indicated by the parametric estimates.

Finally, the different procedures to construct the pointwise and simultaneous CIs for the CDF and the return level function were applied using this data set. The same set *T* = {5, 10, 20, 100, 200, 500, 1000} of return periods as that used in the USGS series was employed here, and a similar analysis was performed. Note that now, the parametric estimates were computed assuming a GEV distribution. Tables 8 and 9 show the CIs constructed with the different methods, presenting a similar information to that in Tables 3–6.

**Table 8:** Lower and upper limits of pointwise (Stand, Perc, Basic and BCa) and simultaneous (Bon-Basic, Bon-BCa and Corr-Bon) CIs for the CDF at quantiles with return periods *T* = {5, 10, 20, 100, 200, 500, 1000}, using a GEV parametric model (top part) and a nonparametric model (bottom part). Ebro river (gaging station at Zaragoza city).

*T* (years)	Stand		Perc		Basic		BCa		Bon-Basic		Bon-BCa		Corr-Bon	
	Low	Up	Low	Up	Low	Up	Low	Up	Low	Up	Low	Up	Low	Up
Parametric fit (GEV)														
5	0.77	0.95	0.71	0.88	0.72	0.88	0.71	0.87	0.69	0.91	0.69	0.89	0.71	0.89
10	0.86	1.00	0.84	0.95	0.84	0.95	0.83	0.95	0.82	0.98	0.82	0.96	0.83	0.97
20	0.92	1.00	0.90	0.99	0.91	0.99	0.89	0.98	0.90	1.00	0.88	0.99	0.90	1.00
100	0.96	1.00	0.95	1.00	0.96	1.00	0.95	1.00	0.96	1.00	0.94	1.00	0.96	1.00
200	0.97	1.00	0.97	1.00	0.98	1.00	0.96	1.00	0.98	1.00	0.96	1.00	0.98	1.00
500	0.98	1.00	0.98	1.00	0.99	1.00	0.97	1.00	0.99	1.00	0.97	1.00	0.99	1.00
1000	0.98	1.00	0.98	1.00	0.99	1.00	0.97	1.00	0.99	1.00	0.97	1.00	0.99	1.00
Nonparametric fit														
5	0.68	0.91	0.69	0.88	0.70	0.89	0.70	0.89	0.67	0.93	0.68	0.90	0.69	0.91
10	0.82	0.99	0.82	0.96	0.83	0.97	0.82	0.96	0.81	1.00	0.80	0.97	0.82	0.99
20	0.90	1.00	0.89	0.99	0.90	1.00	0.88	0.99	0.89	1.00	0.86	0.99	0.89	1.00
100	0.96	1.00	0.95	1.00	0.97	1.00	0.94	1.00	0.97	1.00	0.92	1.00	0.97	1.00
200	0.96	1.00	0.95	1.00	0.97	1.00	0.92	1.00	0.97	1.00	0.92	1.00	0.97	1.00
500	0.97	1.00	0.96	1.00	0.97	1.00	0.93	1.00	0.97	1.00	0.93	1.00	0.97	1.00
1000	0.97	1.00	0.96	1.00	0.98	1.00	0.94	1.00	0.98	1.00	0.94	1.00	0.98	1.00

Numbers are rounded using two significant figures.

**Table 9:** Lower and upper limits of pointwise (Stand, Perc, Basic and BCa) and simultaneous (Bon-Basic, Bon-BCa and Corr-Bon) CIs for the return levels at quantiles with return periods *T* = {5, 10, 20, 100, 200, 500, 1000}, using a GEV parametric model (top part) and a nonparametric model (bottom part). Ebro river (gaging station at Zaragoza city).

*T* (years)	Stand		Perc		Basic		BCa		Bon-Basic		Bon-BCa		Corr-Bon	
	Low	Up	Low	Up	Low	Up	Low	Up	Low	Up	Low	Up	Low	Up
Parametric fit (GEV)														
5	2130	2694	2170	2639	2179	2647	2194	2665	2075	2727	2152	2740	2138	2693
10	2366	3076	2479	3075	2502	3098	2533	3133	2378	3191	2483	3232	2447	3145
20	2506	3445	2725	3519	2734	3528	2798	3588	2618	3659	2737	3674	2666	3600
100	2534	4321	3121	4590	3020	4490	3197	4711	2737	4707	3057	4810	2839	4607
200	2447	4710	3231	5087	3037	4893	3298	5248	2613	5139	3152	5379	2797	5015
500	2258	5236	3350	5805	2943	5398	3419	5976	2274	5689	3239	6241	2584	5528
1000	2065	5644	3416	6351	2831	5766	3486	6549	1937	6081	3301	6941	2289	5904
Nonparametric fit														
5	2177	2761	2203	2700	2210	2708	2232	2741	2097	2793	2192	2812	2160	2745
10	2398	3144	2499	3133	2517	3151	2534	3174	2409	3250	2485	3242	2469	3198
20	2498	3533	2741	3660	2622	3541	2804	3906	2344	3681	2735	3953	2376	3617
100	2176	4132	3150	4130	3912	4892	2939	4130	3912	5110	2832	4130	3912	5102
200	2119	4189	3154	4130	4130	5106	2832	4130	4130	5320	2744	4130	4130	5320
500	2119	4189	3154	4130	4130	5106	2832	4130	4130	5320	2744	4130	4130	5320
1000	2119	4189	3154	4130	4130	5106	2832	4130	4130	5320	2744	4130	4130	5320

Figures included in the Supporting Information (S1)–(S4) Figs show, in a visual way, the information provided in Table 9.

From a hydrological point of view, interesting findings can be deduced from Tables 8 and 9, and from Figures included in the Supporting Information (S1)–(S4) Figs. From Table 8 similar conclusions to those previously described for the USGS series can be obtained. On the other hand, observing the top part of Table 9 (parametric fit), it can be noted that, according to the general conclusions extracted from the simulation study, the best results are obtained, in general, in the columns BCa and Bon-BCa. The CIs corresponding to these columns are slightly wider than those in the other columns. While this might seem a handicap in a first moment, it must be taken into account that the sample size is relatively small. Then, a wider CI could give more reliability to obtain a return level within that interval, for a very long time prediction period (200, 500 or 1000 years). Note that the larger observed value for this series was 4130. This seems to indicate that this value could be exceeded in a relatively large magnitude (assuming that the fit to a GEV distribution is correct), given the increasing shape of the return level function (Fig 4).

Regarding the nonparametric approach, and taking into account the quite small sample size, the best results are obtained with the Basic, Bon-Basic and Corr-Bon methods. Effectively, by looking again at high values for long-term prediction (200, 500 and 1000 years), it is observed that the intervals in the mentioned columns range between 4130 and 5320. As previously pointed out, the maximum observed value in this series was just 4130, so it is likely that this value will be exceeded. The nonparametric approach estimates, however, a maximum value of 5320 (with a 95% of confidence), possibly more realistic than 6941, that is the maximum value predicted using a parametric fit.

## Conclusions

CDF estimation has been revealed as a powerful tool to treat several real questions related with hydrological problems. In this setting, computation of concerning quantities such as the return levels or mean return periods are valuable allied in the study of the natural hazards. The estimation of these functions can be complemented with the construction of CIs, providing information about the reliability of the inference process. This can be carried out computing simultaneous CIs (confidence bands) containing the function of interest with a prescribed high probability. Note that using simultaneous CIs instead pointwise intervals has some practical implications in hydrological applications, because simultaneous CIs include information on model reliability. Hence, they are much more informative than pointwise CIs to assess the goodness of a complete (parametric or nonparametric) model that, subsequently, can be used to make inference or predictions.

The usual process to obtain the confidence bands is based on constructing pointwise CIs in a grid of selected points. However, this procedure can give biased results due to the well-known multiple range testing problem. In the present paper, different approaches to construct pointwise and simultaneous CIs for the CDF (mainly evaluated in large quantiles) and also for the return level function have been compared. Specifically, four methods usually applied to obtain pointwise CIs (standard normal bootstrap, percentile bootstrap, basic bootstrap and BCa methods), and three approaches designed to compute simultaneous CIs (Bonferroni applied to the basic and BCa intervals, and an iterative process combining the Bonferroni and the basic CIs) have been used. These techniques employ bootstrap algorithms. Additionally, parametric and nonparametric methodologies to estimate the functions of interest were applied to calculate the CIs by the different approaches. This research was also devoted to study the behavior of the pointwise methods when they are used to obtain pointwise CIs and simultaneous CIs, and vice versa. The study was completed with the analysis of real river flow data using the previous methods.

In general, it was observed that the BCa method had the best performance among the pointwise procedures considered, while the Bonferroni correction applied to the BCa intervals gave the best results for the simultaneous approaches. On the other hand, it was observed that if a pointwise method is applied to obtain confidence bands, the simultaneous coverage probabilities are far from the nominal value considered. In this sense, modifications of these techniques, using a simple Bonferroni correction has provided better results when the interest is to construct confidence bands. Additionally, nonparametric methods have given better results than misspecified parametric models when the interest function is the CDF and, in this sense, the recommendation is the use of this methodology as a robust procedure very helpful when the real distribution of the data is unknown. However, for return levels it has been found that a nonparametric model led to worse results than a parametric model, and thus for this case we propose using a parametric model selected by means of goodness-of-fit tests.

We have mainly focused on the CDF, because this is the basic function of interest used in the analysis of extreme values in hydrology. However, we have also obtained some results regarding the return levels. The same kind of study could be carried out with other functionals such as the return period, for instance.

## Supporting Information

S1 Text Specific details about parametric and nonparametric density estimations for the Ebro river series. In order to identify significant data features, parametric and nonparametric density estimates were computed. Assuming a parametric model, a GEV distribution was fitted by means of the nsRFA R package [42]. The estimated parameters, using the Lmoments function of the mentioned R package were 1555.73, 613.57 and 0.10 for the location, scale and shape parameters, respectively. Next, by means of the gofGEVtest function of the same package, the Anderson-Darling test (e.g. [26]) was applied to check the goodness-of-fit of these data to a GEV distribution, obtaining *p*-values around 0.2 (depending on the Monte-Carlo replications). Therefore, the assumption of the data following a GEV distribution can not be rejected. Next, the density was nonparametrically estimated, calculating the bandwidth by the plug-in method, directly obtained with the density function of the base R package.
(PDF)

S1 Data Roanoke River database. Annual maximum peak discharges series, measured in cfs, of Roanoke River, VA.
(XLSX)

S2 Data Delaware River database. Annual maximum peak discharges series, measured in cfs, of Delaware River, NJ.
(XLSX)

S3 Data Allegheny River database. Annual maximum peak discharges series, measured in cfs, of Allegheny River, NY.
(XLSX)

S4 Data Arroyo River database. Annual maximum peak discharges series, measured in cfs, of Arroyo River, CA.
(XLSX)

S5 Data Ebro River database. Annual maximum peak discharges series, measured in cumecs, of Ebro River (gaging station at Zaragoza city, Spain).
(XLSX)

S1 Fig Pointwise CIs for the return levels using a GEV parametric model for Ebro river. Pointwise CIs (Stand, Perc, Basic and BCa) for the return levels at quantiles with return periods *T* = {5, 10, 20, 100, 200, 500, 1000} using a GEV parametric model. Ebro river (gaging station at Zaragoza city).
(EPS)

S2 Fig Simultaneous CIs for the return levels using a GEV parametric model for Ebro river. Simultaneous CIs (Stand, Perc, Basic and BCa) for the return levels at quantiles with return periods *T* = {5, 10, 20, 100, 200, 500, 1000} using a GEV parametric model. Ebro river (gaging station at Zaragoza city).
(EPS)

S3 Fig Pointwise CIs for the return levels using a nonparametric model for Ebro river. Pointwise CIs (Stand, Perc, Basic and BCa) for the return levels at quantiles with return periods *T* = {5, 10, 20, 100, 200, 500, 1000} using a nonparametric model. Ebro river (gaging station at Zaragoza city).
(EPS)

S4 Fig Simultaneous CIs for the return levels using a nonparametric model for Ebro river. Simultaneous CIs (Stand, Perc, Basic and BCa) for the return levels at quantiles with return periods *T* = {5, 10, 20, 100, 200, 500, 1000} using a nonparametric model. Ebro river (gaging station at Zaragoza city).
(EPS)
