# Supplementary material for: Comparing Simultaneous and Pointwise Confidence Intervals for Hydrological Processes
Source: PLoS One. 2016 Feb 1;11(2):e0147505. doi: 10.1371/journal.pone.0147505 (PMC4734703; doi:10.1371/journal.pone.0147505)
Supplement: S1 Text — In order to identify significant data features, parametric and nonparametric density estimates were computed. Assuming a parametric model, a GEV distribution was fitted by means of the nsRFA R package [42]. The estimated parameters, using the Lmoments function of the mentioned R package were 1555.73, 613.57 and 0.10 for the location, scale and shape parameters, respectively. Next, by means of the gofGEVtest function of the same package, the Anderson-Darling test (e.g. [26]) was applied to check the goodness-of-fit of these data to a GEV distribution, obtaining p-values around 0.2 (depending on the Monte-Carlo replications). Therefore, the assumption of the data following a GEV distribution can not be rejected. Next, the density was nonparametrically estimated, calculating the bandwidth by the plug-in method, directly obtained with the density function of the base R package. (PDF) [file pone.0147505.s001.pdf]

S1 Text

Specific details about parametric and nonparametric density estimations for the Ebro river series. In order to identify significant data features, parametric and nonparametric density estimates were computed. Assuming a parametric model, a GEV distribution was fitted by means of the `nsRFA` R package [42]. The estimated parameters, using the `Lmoments` function of the mentioned R package were 1555.73, 613.57 and 0.10 for the location, scale and shape parameters, respectively. Next, by means of the `gofGEVtest` function of the same package, the Anderson-Darling test (e.g. [26]) was applied to check the goodness-of-fit of these data to a GEV distribution, obtaining p-values around 0.2 (depending on the Monte-Carlo replications). Therefore, the assumption of the data following a GEV distribution can not be rejected. Next, the density was nonparametrically estimated, calculating the bandwidth by the plug-in method, directly obtained with the `density` function of the `base` R package.
